# An Artificial Intelligence-Based Alarm Strategy Facilitates Management of Acute Myocardial Infarction

**DOI:** 10.3390/jpm11111149

**Published:** 2021-11-04

**Authors:** Wen-Cheng Liu, Chin Lin, Chin-Sheng Lin, Min-Chien Tsai, Sy-Jou Chen, Shih-Hung Tsai, Wei-Shiang Lin, Chia-Cheng Lee, Tien-Ping Tsao, Cheng-Chung Cheng

**Affiliations:** 1 Division of Cardiology, Department of Internal Medicine, Tri-Service General Hospital, National Defense Medical Center, Taipei 114, Taiwan; skyb1983@hotmail.com (W.-C.L.); littlelincs@gmail.com (C.-S.L.); wslin545@ms27.hinet.net (W.-S.L.); 2 Medical Technology Education Center, School of Medicine, National Defense Medical Center, Taipei 114, Taiwan; xup6fup0629@gmail.com; 3 School of Public Health, National Defense Medical Center, Taipei 114, Taiwan; 4 Graduate Institute of Life Sciences, National Defense Medical Center, Taipei 114, Taiwan; 5 Graduate Institute of Physiology and Biophysics, National Defense Medical Center, Taipei 114, Taiwan; mctsai6108@gmail.com; 6 Department of Emergency Medicine, Tri-Service General Hospital, National Defense Medical Center, Taipei 114, Taiwan; syjou.chen@gmail.com (S.-J.C.); tsaishihung@yahoo.com.tw (S.-H.T.); 7 Department of Medical Informatics, Tri-Service General Hospital, National Defense Medical Center, Taipei 114, Taiwan; lcgnet@gmail.com; 8 Division of Colorectal Surgery, Department of Surgery, Tri-Service General Hospital, National Defense Medical Center, Taipei 114, Taiwan; 9 Division of Cardiology, Heart Centre, Cheng Hsin General Hospital, Taipei 114, Taiwan

**Keywords:** artificial intelligence, acute myocardial infarction, alarm system, deep learning, electrocardiogram

## Abstract

(1) Background: While an artificial intelligence (AI)-based, cardiologist-level, deep-learning model for detecting acute myocardial infarction (AMI), based on a 12-lead electrocardiogram (ECG), has been established to have extraordinary capabilities, its real-world performance and clinical applications are currently unknown. (2) Methods and Results: To set up an artificial intelligence-based alarm strategy (AI-S) for detecting AMI, we assembled a strategy development cohort including 25,002 visits from August 2019 to April 2020 and a prospective validation cohort including 14,296 visits from May to August 2020 at an emergency department. The components of AI-S consisted of chest pain symptoms, a 12-lead ECG, and high-sensitivity troponin I. The primary endpoint was to assess the performance of AI-S in the prospective validation cohort by evaluating F-measure, precision, and recall. The secondary endpoint was to evaluate the impact on door-to-balloon (DtoB) time before and after AI-S implementation in STEMI patients treated with primary percutaneous coronary intervention (PPCI). Patients with STEMI were alerted precisely by AI-S (F-measure = 0.932, precision of 93.2%, recall of 93.2%). Strikingly, in comparison with pre-AI-S (N = 57) and post-AI-S (N = 32) implantation in STEMI protocol, the median ECG-to-cardiac catheterization laboratory activation (EtoCCLA) time was significantly reduced from 6.0 (IQR, 5.0–8.0 min) to 4.0 min (IQR, 3.0–5.0 min) (*p* < 0.01). The median DtoB time was shortened from 69 (IQR, 61.0–82.0 min) to 61 min (IQR, 56.8–73.2 min) (*p* = 0.037). (3) Conclusions: AI-S offers front-line physicians a timely and reliable diagnostic decision-support system, thereby significantly reducing EtoCCLA and DtoB time, and facilitating the PPCI process. Nevertheless, large-scale, multi-institute, prospective, or randomized control studies are necessary to further confirm its real-world performance.

## 1. Introduction

Acute coronary syndrome, consisting of ST elevation myocardial infarction (STEMI) and non-ST elevation myocardial infarction (NSTEMI) based on electrocardiogram (ECG) presentations, refers to a spectrum of conditions that abruptly cause an unmet need for coronary blood supply to the myocardium [1,2,3]. Primary percutaneous coronary intervention (PPCI) is currently the standard reperfusion therapy for STEMI. [4]. Door-to-balloon (DtoB) time, which is defined as the interval between emergency department (ED) arrival and the first balloon inflation during PPCI, should be less than 90 min and is regarded as an important metric for PPCI [2,4,5].

Several factors lead to prolonged DtoB time, which can be categorized into patient, hospital, healthcare practice, physician, or other characteristics [6]. Any delay in DtoB time contributes to a higher risk of adverse outcomes. A longer delay (>90 min) in DtoB time was associated with significantly higher overall mortality compared with a shorter delay (≤90 min) [7]. Delayed PPCI independently contributes to long-term risks for heart failure rehospitalizations or outpatient visits [8]. Additionally, a longer DtoB time significantly increased cardiovascular-related health costs [9]. In contrast, a shorter DtoB time provided better blood flow in the infarct-related artery, enhanced restoration of the left ventricular function, and lowered recurrent MI and mortality [6,10,11]. The term “time is muscle” strongly emphasizes timely care for AMI patients [12]. In this regard, an ESC guideline even recommended that DtoB time should be shortened to within 60 min after STEMI diagnosis in PPCI-capable canters [1]. All of the evidence indicates the critical role of DtoB time in the treatment of STEMI patients.

To shorten DtoB time, intensive strategies, including: simplified cardiac catheterization laboratory activation (CCLA) by emergency physicians with a single call to a central page operator; recommending a time of less than 20 min from arrival to the CCL to staff being paged; establishing an on-site cardiologist; and providing real-time data feedback have been proposed [13]. Through these strategies, the DtoB time was reduced from 96 to 71 min with a corresponding increase in the rate of achieving DtoB time within 90 min from 47% to 80% in Taiwan [14,15]. Importantly, reducing the time between obtaining an ECG and CCLA (EtoCCLA), which depends on a prompt diagnosis of STEMI, appears to be a key factor in shortening DtoB time [6,16]. The emergency department is a challenging environment where medical errors often occur due to intense time pressures, heavy workloads, and harried staff during busy working hours [17,18,19,20]. The rate of misdiagnosis of AMI resulting from misinterpretation of ECG at first medical contact ranges from 2 to 30%, which results in subsequent adverse outcomes [21,22]. Therefore, systemic processes to assist and alert front-line physicians at an ED in detecting AMI by ECG effectively may help to reduce the EtoCCLA time and further shorten the DtoB time.

The current artificial intelligence (AI) revolution that started with a deep-learning model (DLM) has provided us with an unprecedented opportunity to improve the healthcare system. Recently, cardiologist-level DLMs for detecting AMI have been developed [23,24,25]. We demonstrated that our DLM exhibits better performance than those of the current experts in detecting AMI, with a diagnostic capacity of 98.4% and specificity of 96.9% for STEMI detection [25]. In the present study, we further evaluated whether the incorporation of DLM into a diagnostic strategy at an ED facilitates AMI diagnosis and care processes. Accordingly, we set up a tailored active alarm system to provide information regarding patients with AMI via smartphones. We hypothesize that the proposed strategy may minimize EtoCCLA time and further reduce DtoB time for STEMI patients.

## 2. Method

### 2.1. Study Design and Setting

The study was a single-center, prospective-validation, and before-and-after study conducted in the Tri-Service General Hospital, Taipei, Taiwan to develop an artificial intelligence-based alarm strategy (AI-S) and evaluate the performance of AI-S for AMI detection. The study enrollment took place at the ED from August 2019 to August 2020. The study protocol was approved by the Institutional Review Board of the Tri-Service General Hospital, National Defense Medical Center (IRB No. C202005055), in accordance with the ethical guidelines of the Declaration of Helsinki of the World Medical Association.

### 2.2. Study Population for AI-S Development

All adult patients (18 years or older) presenting to the ED receiving a 12-lead ECG acquired in the supine position were included. Patients with an existing permanent pacemaker, those with a clinical STEMI ECG without PPCI, and those who died at the ED without PPCI were all excluded. The AI-S was developed by a strategy development cohort and validated by a prospective validation cohort. The development cohort from August 2019 to April 2020 included 25,002 ED visits. The validation cohort from May to August 2020 included 14,296 ED visits, as shown in Figure S1. A total of 13 ED visits without PPCI but identified by AI-S in the validation cohort were excluded from the primary analysis, but we assumed them to be false predictions in a sensitivity analysis.

### 2.3. Definition of AMI, DtoB Time Metrics and STEMI Protocol

AMI includes symptoms of myocardial ischemia, ECG presentation, and elevated high-sensitivity troponin I (hsTnI) (above the 99th percentile of the upper reference limit of healthy individuals), which included both STEMI and NSTEMI [1,2,3]. In this study, the diagnosis of STEMI and NSTEMI was confirmed by coronary angiogram. The DtoB time is denoted as the time between the arrival of a STEMI at ED until a balloon was inflated in the occluded culprit coronary artery [3,5]. The DtoB time (goal, ≤90 min) was divided into four clinically relevant quality indicators: door-to-ECG time (DtoE) (goal, ≤10 min), EtoCCLA (goal, ≤10 min), CCLA-to-CCL door (CCLAtoCCLD) time (goal, ≤35 min), and CCLD-to-balloon time (CCLDtoB) (goal, ≤35 min). The details of the STEMI protocol for PPCI are presented in the Supplementary Material and Figure S2.

### 2.4. Data Collection

ECG recordings were collected using a Philips 12-lead ECG machine (PH080A), and the ECG signal was recorded in a digital format. The sampling frequency was 500 Hz, with 10 s recorded in each lead. Patient characteristics, including chief complaints, sex, age, body mass index, blood pressure, medical histories, and laboratory tests, were collected from our electronic medical record (EMR). The door time was recorded at triage and uploaded in the EMR. The ECG time was extracted from the electronic copy of the ECG stored in the Picture Archiving and Communication System. The CCLA time and CCLD time were recorded at the CCL. The balloon time was recorded from the angiographic image of thrombus aspiration or balloon dilatation in the PACS system.

### 2.5. AI-S

#### 2.5.1. First Part of AI-S: The Set-Up of AI-S

All obtained ECGs were uploaded in real time to the AI-S platform to perform AMI autodiagnosis. The STEMI and NSTEMI scores predicted by AI-S were calculated on a background server, which was developed by our previous study [25]. The details of the AI-enabled ECG algorithm are presented in the Supplementary Material and Figure S3. Each ECG obtained a STEMI and a NSTEMI score ranging from 0 to 1 within 10 s and was stored in our EMR. Meanwhile, triage provided the symptom assessment, and the core laboratory immediately uploaded the laboratory data. The AI-S incorporated chest pain symptoms, a 12-lead ECG, and hsTnI to produce a prediction score for AMI diagnosis. The rules of AI-S were updated if the follow-up ECG and hsTnI were evaluated as producing higher prediction scores of STEMI or NSTEMI than the initial data as shown in Figure S4.

#### 2.5.2. Second Part of AI-S: Automatic Active Alarm System with Notification by Short Message

Once the AI-S indicated STEMI or NSTEMI, a warning message was immediately triggered and sent to the front-line physician in charge of patient at the ED and the on-duty cardiologist. Notifications appeared in the recipient’s on-duty smartphone message system for prompt attention, as shown in Supplementary Materials and Figure S5. The short message was only sent once for the earliest triggering rule, and was not triggered by negative samples after multiple background calculations by AI.

### 2.6. Study Outcomes

The primary analysis was to evaluate the performance of AI-S for STEMI and NSTEMI detection by F-measure, precision, recall, and stratified analyses in the prospective validation cohort. We analyzed the specific features to better identify AMI. The secondary analysis evaluated each component of DtoB time before and after AI-S implantation. We arbitrarily chose patients during the same period before the AI-S (from May to Aug in 2018 and in 2019) as the control group and patients in the prospective cohort after the AI-S (from May to August in 2020) as the intervention group. The non-transferred STEMI cases meeting the DtoB time within 90 min were analyzed and compared. One-year major adverse cardiac events (MACEs) after PPCI including all-cause mortality, heart failure hospitalization, and nonfatal MI after PPCI, before and after AI-S implementation were evaluated.

### 2.7. Statistical Analysis

The study cohort was divided into a strategy development cohort and a prospective validation cohort. We presented their characteristics as the means with standard deviations or medians with interquartile ranges (IQR), numbers of patients, or percentages where appropriate. They were compared using either Student’s *t*-test or the chi-square test, as appropriate. The statistical analysis was performed using R software version 3.4.4. (R Foundation for Statistical Computing, Vienna, Australia). All analyses were based on ED visits. A significance level of *p* < 0.05 throughout the analysis was used.

To evaluate and define the most accurate AI-S, confusion matrixes were adopted to calculate the precision (positive predictive value), recall (sensitivity), and specificity of each diagnostic strategy. The receiver operating characteristic (ROC) curve, precision–recall ROC (PRROC) curve, and the corresponding area under the curve (AUC) were presented. The operating points of AI-S were decided by the maximum of the F-measure in the strategy development cohort, and they were applied in our AI-S for the prospective validation cohort. The F-measure is a global indicator to integrate recall and precision, which can be calculated as follows: F-measure = (2 × precision × recall)/(precision + recall).

## 3. Results

Baseline characteristics of the cohorts was shown in Supplementary Materials and Table S1.

### 3.1. The Development of AI-S

**Figure 1:**
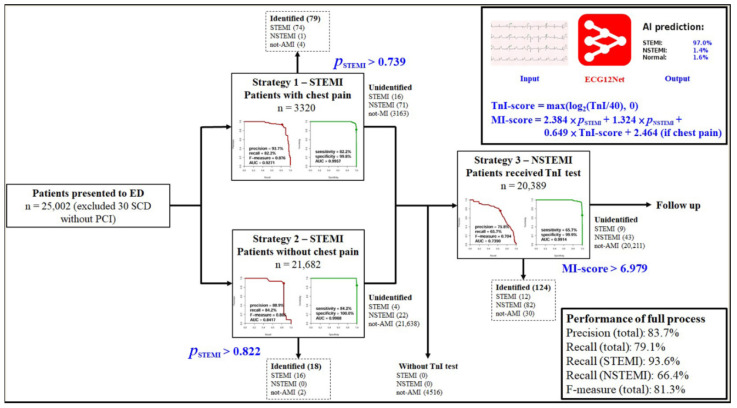
The workflow and development of the AI-based alarm strategy. All visits were divided into two groups with or without chest pain for STEMI detection. The remaining visits with hsTnI were used for subsequent NSTEMI detection. The AUROC curve and PRROC curve were generated by the highest probability of STEMI/NSTEMI prediction by our AI and hsTnI.

Figure 1 summarizes the development of AI-S. Initially, at triage, patients with a chief complaint of chest pain were included in strategy 1 for 3320 patients, whereas patients without a chief complaint of chest pain were included in strategy 2 for 21,682 patients. Both strategies were only based on the STEMI score predicted by AI via ECG. Strategy 1, with an operating point of 0.739, achieved STEMI prediction with an AUC of 0.996, a corresponding sensitivity of 82.2%, and specificity of 99.8%. Strategy 2, with an operating point of 0.822, achieved STEMI prediction with an AUC of 0.999, a corresponding sensitivity of 84.2%, and specificity of 100.0%. The remaining cases received the following MI-score screening, which was generated by logistic regression analysis for 20,389 patients with hsTnI values. Strategy 3 achieved NSTEMI prediction with an AUC of 0.991, a corresponding sensitivity of 65.7% and specificity of 99.9%. For NSTEMI, an MI score greater than 6.979 was triggered. All analyses in this part directly used the highest STEMI and NSTEMI scores, and finally, the AI-S provided an F-measure of 0.813 in the strategy development cohort. We subsequently used this AI-S in a prospective validation cohort.

### 3.2. The Performance of AI-S for STEMI Detection

**Figure 2:**
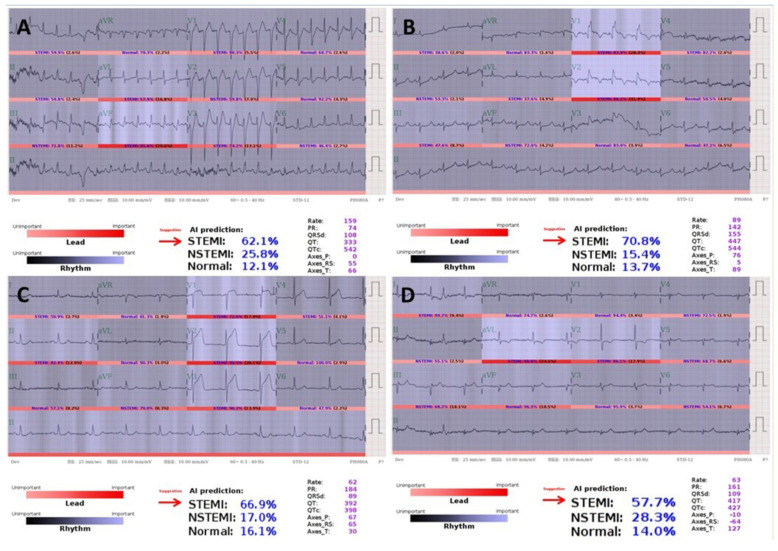
STEMIs for which alarms failed to be raised by the AI-based alarm strategy. (**A**,**B**) are both post-resuscitation ECGs with STEMI prediction scores of 62.1% and 70.8%, respectively. (**C**) Hyperacute T presentation with prompt initiation of PPCI with a STEMI prediction score of 66.9%. (**D**) Inferior wall STEMI with deep Q in III, aVF and reciprocal ST-T change in I, aVL, V2 with STEMI prediction score of 57.7%.

Prospective validation of AI-S for AMI detection is shown in Table 1. During the prospective study period, 55 of 59 (93.2%) STEMI cases were notified, as expected by strategies 1 and 2, but 4 (6.8%) of them were notified by strategy 3 later, as shown in Figure 2. Both Figure 2A,B were out-of-hospital cardiac arrests with return of spontaneous circulation. Figure 2C received PPCI. Figure 2D was initially considered as NSTEMI by the front-line physician. Nevertheless, all four ECGs indicated STEMI by AI but did not reach the operating threshold for AI-S, with still-high STEMI prediction scores of 0.621, 0.708, 0.669, and 0.577, respectively.

### 3.3. The Performance of AI-S for NSTEMI and Not-AMI Detection

For NSTEMI detection, AI-S showed a lower sensitivity of 62.1% but a high specificity of 99.96%. For not-AMI detection, only 4 of 14,171 cases with alarms raised by strategies 1 and 2, initiated PPCI, as shown in Figure S6. The final diagnosis was as follows: both Figure S6A,B were diagnosed with acute perimyocarditis. Figure S6C was takotsubo cardiomyopathy. Figure S6D was a coronary spasm with a patent coronary artery.

### 3.4. Delayed/Misdiagnosed Cases by Front-Line Physicians

**Figure 3:**
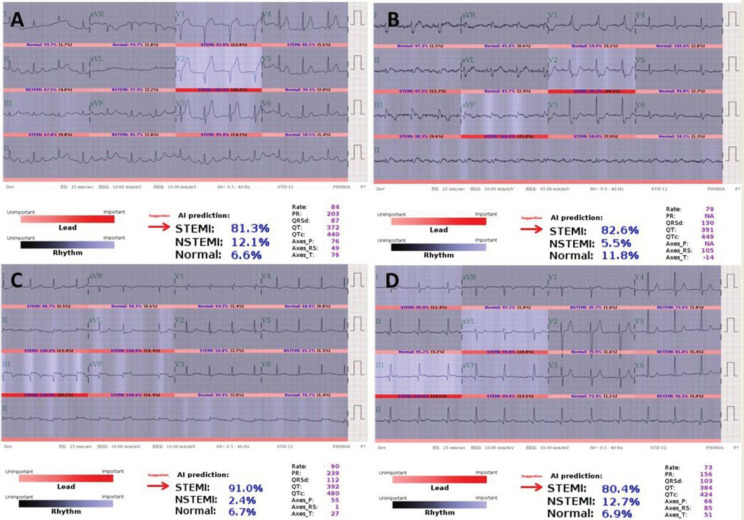
STEMIs for which alarms were raised by the AI-based alarm strategy but for which diagnosis by front-line physicians were delayed. (**A**) A 36-year-old tall man awaiting chest X-ray to rule out pneumothorax. (**B**) An 82-year-old woman presenting with left shoulder pain initially. (**C**) A 75-year-old woman presenting with acute epigastric pain, nausea, and dizziness awaiting an X-ray to rule out a perforated peptic ulcer. (**D**) A 54-year-old man with a chronic kidney disease awaiting labs to rule out hyperkalemia.

During the prospective study period, four of 59 STEMI (6.8%) cases were delayed diagnoses by front-line physicians, as shown in Figure 3. The detailed description of these four cases was shown in Supplementary Materials. The first three cases received PPCI and still met the DtoB time because the on-duty cardiologist actively confirmed the ECG and activated the CCL with the aid of AI-S. The final case was missed and delayed PPCI by both the ED physician and cardiologist.

### 3.5. The DtoB Time Metrics before and after AI-S Implantation

**Figure 4:**
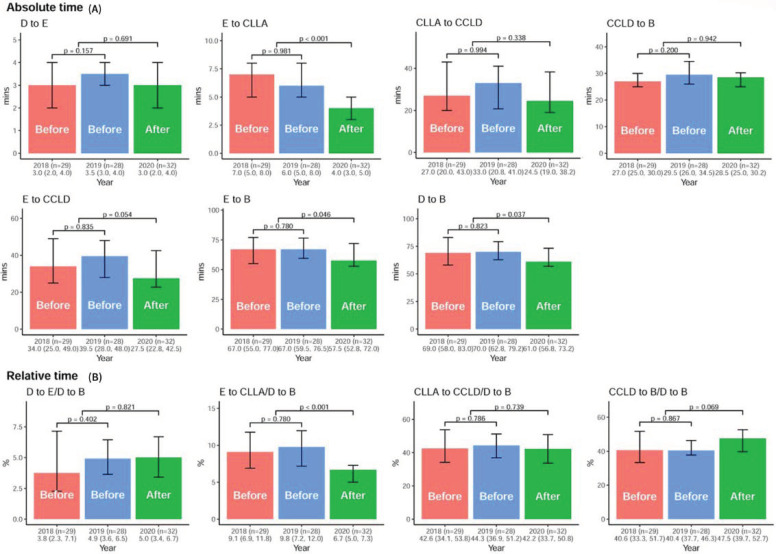
The DtoB time metrics before and after the AI-based alarm strategy. (**A**) The comparison of the median DtoE, EtoCCLA, CCLAtoCCLD, CCLDtoB, EtoCCLD, EtoB, and DtoB times before and after AI-S implantation. (**B**) The relative time ratio between each time component over DtoB time.

The time metric of each component of DtoB time including DtoE, EtoCCLA, CCLAtoCCLD, CCLDtoB time, and the composite interval with EtoCCLD, EtoB, and DtoB time for cases meeting the DtoB time within 90 min, were analyzed before and after AI-S implantation, as shown in Table S2 and Figure 4. After AI-S, the median EtoCCLA time was significantly reduced from 6.0min (IQR, 5.0–8.0 min) to 4.0 min (IQR, 3.0–5.0 min, *p* < 0.01). Furthermore, the median DtoB time was significantly shortened from 69 min (IQR, 61.0–82.0min) to 61 min (IQR, 56.8–73.2 min, *p* = 0.037). For the relative time ratio between each time component to DtoB time, only EtoCCLA time was significantly shortened in the AI-S group, indicating the impact of AI-S on EtoCCLA time. The one-year MACEs after PPCI showed no significant difference before and after AI-S in these STEMI patients as shown in Table S3.

### 3.6. The Performance of AI-S in Different Groups and Clinical Features

**Figure 5:**
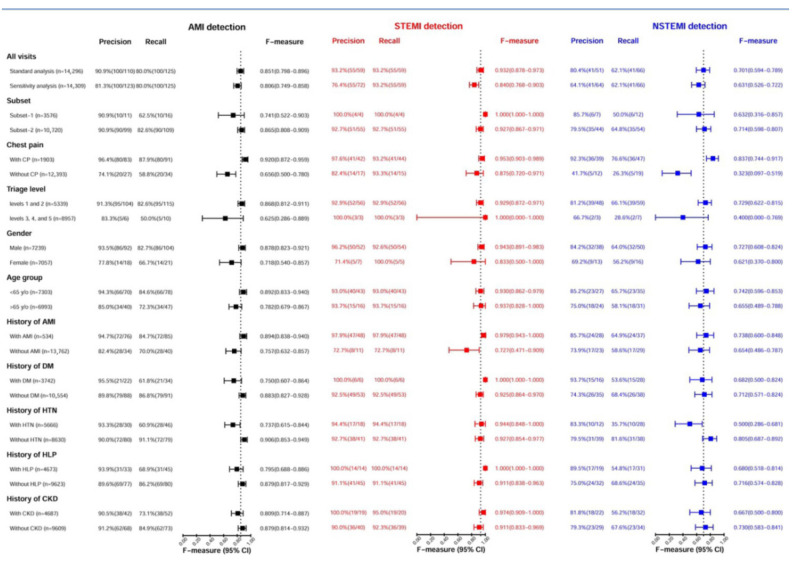
Stratified analyses for the performance of AI-based alarm strategy in AMI, STEMI, and NSTEMI in the prospective cohort. The 95% CI of F-measures was calculated based on 10,000 bootstrapping experiments. For the standard analysis, the F-measure of AMI detection was 0.851 (95% CI: 0.798–0.896), the F-measure of STEMI detection was 0.932 (95% CI: 0.878–0.973), and the F-measure of NSTEMI detection was 0.701 (95% CI: 0.594–0.789).

The performance of AI-S using the F-measure was analyzed to evaluate the impact of clinical features on AMI detection, as shown in Figure 5. The F-measure of AMI detection using this integrated AI-S was 0.851 (95% CI: 0.798–0.896) for the standard analysis and 0.806 (95% CI: 0.749–0.858) for the sensitivity analysis. For STEMI detection, the F-measure was 0.932 (95% CI: 0.878–0.973), with a precision of 93.2% and recall of 93.2%. For NSTEMI detection, the F-measure was 0.701 (95% CI: 0.594–0.789). Moreover, striated analyses showed that AI-S provides better performance in patients with a history of AMI, diabetes, and hyperlipidemia (F-measure = 0.979, 1.0, 1.0) for STEMI detection. Furthermore, AI-S had a better performance in patients with chest pain than in those without chest pain (F-measure = 0.920 vs. 0.656) for AMI detection, and this superiority was primarily for NSTEMI detection (F-measure = 0.837 vs. 0.323).

## 4. Discussion

We established an AI-S to early identify patients with AMI and promptly initiate team-based PPCI for STEMI (graphical abstract). Our AI-S achieved an F-measure of 0.932 (95% CI: 0.878–0.973) with a precision of 93.2% and a recall of 93.2% in STEMI detection. With the AI-S, the EtoCCLA, EtoB, and DtoB times were significantly reduced compared with those without AI-S. Further stratified analyses revealed that AI-S performed better in STEMI detection among patients with a history of AMI, diabetes, or hyperlipidemia than those without. For NSTEMI detection, the symptoms of chest pain further enhanced the diagnostic power of AI-S. AI-S obviously provides extraordinary decision support for front-line physicians to manage patients with AMI at the ED.

In achieving a DtoB time ≤ 90 min, the rate-determining step was DtoCCLA time, composed of DtoE and EtoCCLA time [16,26]. Although a DtoE time ≤ 10 min, as recommended in the majority of national guidelines, was notably achieved in our clinical practice, the critical role of EtoCCLA time is rarely addressed. The causes of delayed EtoCCLA time include incorrect interpretation of the ECG in the context of the patient’s symptoms, and complicated patients with atypical presentations or multiple comorbidities. To improve EtoCCLA time, direct activation of CCL by ED physicians or an available on-site cardiologist is recommended [13]. Direct activation of CCL by ED physicians increased the rate of false-positive activation to 9–39%, resulting in unnecessary procedures and medical resources, while an on-site cardiologist is not always available, especially in regional hospitals, and rural or remote areas [27,28,29]. The AI-S provides both diagnostic quality and cost effectiveness with fairly feasible, noninvasive, and inexpensive characteristics.

Recent innovative strategies have been developed to shorten EtoCCLA time by facilitating the availability of ECG images to on-duty cardiologists. Chao et al. used the smartphone to transmit ECG images to working groups of ED physicians and cardiologists with reduced EtoCCLA time from 28.3 to 17.6 min [30]. Chen et al. implemented a tele-ECG triage system, which is read by ED physicians and transmits the probable ECG to cardiologists, with a reduced EtoCCLA time from 25 to 7 min [31]. Zhang et al. integrated an ECG server with a short message to transmit ED physician-read ECG images to cardiologists, which could reduce DtoB time [32]. Despite the promising effects of these strategies, large numbers of false positive alarms and false negative detections by ED physicians inevitably occur. AI-S provides precise and timely notification by reducing unnecessary human errors.

Our AI-S integrated information on chest pain symptoms, 12-lead ECG, and hsTnI, serving as a promising diagnostic support tool to alert physicians for the management of patients with high-risk NSTEMI. The application of hsTnI to the detection of NSTEMI patients achieves a negative predictive value (NPV) of 99% and a positive predictive value (PPV) of 70–75% [3]. Importantly for the detection of NSTEMI requiring PCI, the AI-S also demonstrated a high NPV of 99.8% and PPV of 80.4%, which helps physicians rule out low-risk populations. Moreover, chest pain presentation enhances the diagnostic power of AI-S for the detection of NSTEMI (F-measure = 0.837 vs. 0.323). These results highlight the roles of AI-S in the diagnosis of acute coronary syndrome in addition to STEMI.

Delayed or misdiagnosis of STEMI ECG significantly contributed to delayed PPCI. A recent study reported that the median EtoCCLA time requires 8 min in ED physicians, even with typical STEMI presentation on ECGs [16]. Importantly, the ECG presentations of STEMI-equivalent or subtle occlusion greatly prolonged the EtoCCLA time. Our AI-S had extraordinary diagnostic power to differentiate these high-risk STEMI ECGs as shown in Figure 3, which was verified in this study. This fact partly explains the reduced EtoCCLA time in our AI-S.

False CCLA remains a critical issue in the management of AMI. Previous studies revealed a higher rate of false positive diagnosis or activation of STEMI from 9.0% to 39%. The main reason for false CCLA was the misinterpretation of ECG presentations due to pre-existing or secondary pathologic ECG abnormalities [27]. In our study, four false alarms (4/59, 6.8%) occurred, as shown in Figure S5, resulting only from secondary pathologic ECG abnormalities misdetected as STEMI by AI-S. The capacity of AI-S to exclude pre-existing ECG abnormalities as STEMI mimics partly elucidates the low false alarm rate in our system [25].

Owing to the notable diagnostic support and alarm system in STEMI detection at ED, AI-S has several potential clinical applications in the future. First, it may replace single call activation to activate the whole CCL team, including the ED physician, on-duty cardiologist, and CCL staff, once the STEMI ECG is obtained. Second, ECG machines in ambulances can be modified to facilitate telemedicine, which may reduce physician resources and loading and provide early CCLA to shorten DtoB time. Third, it may be applied in rural and remote areas and places lacking experts for ECG interpretation to promote diagnostic accuracy, thereby facilitating timely management [28,29].

## 5. Limitations and Strengths

The limitations of the present study should be mentioned. First, this was a prospective and before-and-after study rather than a randomized controlled trial. Potential confounding variables that might affect DtoB time may not be controlled. Second, the studied patients were enrolled from only one academic medical center, although the diagnosis and management of AMI was based on guidelines. Multicenter validation is needed to confirm the application of this study. Third, the implementation of AI-S is a new tool in the STEMI protocol in 2020, which may cause a Hawthorne effect [33]. Nevertheless, it is worth introducing the system to strengthen the decrease in DtoB time, attributed to the improvement in EtoCCLA time. Fourth, although DtoB time is distinctly a key indicator of quality performance, whether this decreased DtoB time can be translated into improved outcomes is still a subject for ongoing research. Fifth, further investigation as to whether other patients’ characteristics could affect the performance of AI-S for the AMI detection is needed. Finally, despite the remarkable results obtained with STEMI detection, AI-S can only serve as a diagnostic support tool. The diagnostic decision is still made by front-line physicians or on-duty cardiologists. The strength of the present study is that DLM has been shown to achieve expert-level performance when large annotated datasets are available. Although clinical decision-making requires multidisciplinary information, leading to hindrances in the application of DLM to real-world practice, the success of our AI-S was to integrate real-time information on chest pain symptoms from triage and cardiac enzyme data from the central laboratory. Moreover, although previous innovative alarming systems activated by frontline physicians have been reported, they require large amounts of human resources. Our automatic AI-S integrated DLM with a short message service to offer timely and precise alarming to on-duty cardiologists, and helps to reduce DtoB time without delay and, importantly, without increasing any workload, which provides a better strategy for AMI care.

## 6. Conclusions

We developed a novel AI-S to facilitate the diagnosis and time to treatment of AMI at the ED. AI-S had an exceptional diagnostic power for STEMI. With the aid of AI-S, alarms were raised for STEMI in a timely, simultaneous and precise manner for both the ED physician and on-duty cardiologist to activate CCL, which significantly shortens the EtoCCLA and DtoB time. Additionally, our AI-S serves as an excellent diagnostic supporting system to rule out low-risk populations of AMI. Further randomized controlled trials are needed to confirm the performance of the AI-S for AMI detection and its impacts on the reduction of DtoB time and clinical outcomes.

## Figures and Tables

**Table 1:** The performance of acute myocardial infarction detection by an AI-based alarm strategy in the prospective validation cohort.

	Prospective Validation Cohort			
	STEMI	NSTEMI	not-AMI	Excluded
All dataset				
Alarmed	59 (100.0%)	41 (62.1%)	10 (0.1%)	13 (100.0%)
Strategy-1	41	0	1	2
Strategy-2	14	1	3	11
Strategy-3	4	40	6	0
Unalarmed	0 (0.0%)	25 (37.9%)	14,161 (99.9%)	0 (0.0%)
Subset-1				
Alarmed	4 (100.0%)	6 (50.0%)	1 (<0.1%)	3 (100.0%)
Strategy-1	3	0	0	1
Strategy-2	1	1	0	2
Strategy-3	0	5	1	0
Unalarmed	0 (0.0%)	6 (50.0%)	3559 (>99.9%)	0 (0.0%)
Subset-2				
Alarmed	55 (100.0%)	35 (64.8%)	9 (0.1%)	10 (100.0%)
Strategy-1	38	0	1	1
Strategy-2	13	0	3	9
Strategy-3	4	35	5	0
Unalarmed	0 (0.0%)	19 (35.2%)	10,602 (99.9%)	0 (0.0%)

Abbreviations: AI, artificial intelligence; AMI, acute myocardial infarction; STEMI, ST-elevation myocardial infarction; NSTEMI, non-ST-elevation myocardial infarction.

## Data Availability

The data presented in this study are available on request from the corresponding author.

## Supplementary Materials

The following are available online at https://www.mdpi.com/article/10.3390/jpm11111149/s1, Figure S1: Flow diagram of the generation of strategy development and prospective validation cohorts, Figure S2: Comparison of the STEMI protocol before and after AI-based alarm strategy, Figure S3: Architecture of AI-enable ECG algorithm, Figure S4: The rules of AI-based alarm strategy, Figure S5: Screenshot of smartphone notification messages triggered by AI-based alarm strategy, Figure S6: Not-AMI ECGs with initial detection as STEMI by AI-based alarm strategy, Table S1: Corresponding characteristics in development and prospective validation cohorts, Table S2: Baseline characteristics of study participants before and after AI-S implantation, Table S3: One-year outcomes of study participants after primary percutaneous coronary intervention with door-to-balloon time ≤ 90min.

## Abbreviations

STEMI, ST elevation myocardial infarction; NSTEMI, non-ST elevation myocardial infarction; ECG, electrocardiogram; PPCI, Primary percutaneous coronary intervention; DtoB, Door-to-balloon; ED, emergency department; CCLA, cardiac catheterization laboratory activation. DLM, deep learning model; AI-S, artificial intelligence-based alarm strategy; DtoE, door-to-ECG; EtoCCLA, ECG-to-CCLA; CCLAtoCCLD, CCLA-to-CCL door; CCLDtoB, CCLD-to-balloon; EMR, electronic medical record; MACE, major adverse cardiac event; IQR, interquartile ranges; ROC, the receiver operating characteristic curve; PRROC, precision-recall ROC curve; AUC, area under the curve.
