# Supplementary material for: An Artificial Intelligence-Based Alarm Strategy Facilitates Management of Acute Myocardial Infarction
Source: J Pers Med. 2021 Nov 4;11(11):1149. doi: 10.3390/jpm11111149 (PMC8623357; doi:10.3390/jpm11111149)
Supplement: Supplementary file 1 [file jpm-11-01149-s001.zip › Supplementary material_JPM.pdf]

An artificial intelligence-based alarm strategy facilitates management of acute myocardial infarction

Supplementary Appendix 1. Methods

The STEMI protocol: the study setting

Tri-Service General Hospital is an academic medical center in Neihu District, Taipei, Taiwan with 1,800 beds, an approximately 1,500,000 annual outpatient department volume, three cardiac catheterization laboratories and on-site cardiothoracic surgery. An average of 2000 diagnostic coronary angiograms and 1200 percutaneous coronary interventions are performed each year. Our facility has eight on-duty interventional cardiologists, each performing an average of 200 cardiac catheterizations each year.

The STEMI protocol: quality control

Our government audits the quality of care for acute coronary syndrome at 4-year intervals, according to the regulations for emergency care capacity accreditation (ECCA).[34] Four quality indicators are defined, including the target rates of DtoE time <10 min, DtoB time ≤90 min, door-to-cardiac enzyme time <120 min, and prescription of dual antiplatelet agents. For DtoB time, the achievement percentage should be more than 75%. STEMI patients enrolled in DtoB time quality metrics were excluded as follows: cardiac arrest within 24 hours of presentation, cardiogenic shock or respiratory failure requiring mechanical circulatory or ventilator support, evident fatal or major bleeding requiring blood transfusion, transfer from another facility, no STEMI on initial ECG, failed PCI or referral for coronary artery bypass graft surgery, patients under clinical trial, and patients refusing PPCI and signing refusal of treatment consent.

These quality indicators have been collected by our quality control center since 2008 with a monthly analysis of cases that do not fulfill any 1 of the 4 quality indicators. Since the first ECCA audit in 2009, the best practice strategies suggested by the DtoB Alliance have been implemented and modified into our protocol, including

a 12-lead ECG obtained during triage for patients with chest pain, cardiologist within the hospital all times, use of computerized provider order entry with a pre-constructed order set to streamline ordering of tests, procedures, and medication administration after CCL activation, storage of STEMI drug packs at ED, CCL staff arrival to CCL within 30 min after being paged, quick patient transportation from the ED to the catheterization room and real-time case feedback.[13,35,36]

The STEMI protocol: PPCI process

In our hospital, an on-duty cardiologist stays at the duty room of the CCL, which is located one floor above the ED and is available for 24 hours. CCL is activated by cardiologists with a single call to the central page. Once STEMI presents, ED physicians call for on-duty cardiologists, who are required to arrive at the ED within 5 min. CCL activation is mandated by on-duty cardiologists after brief evaluation of the patient's condition and confirmation of STEMI ECG. The STEMI team for PPCI is made up of an interdisciplinary group including cardiologist, cardiology fellow, CCL staff, ED physicians, and ED nurses. We hold a monthly combined meeting to examine all STEMI cases in the previous month. **Figure S2** outlines the steps involved at each time interval of the STEMI protocol before and after AI-S.

Artificial intelligence-enable ECG algorithm[25]

Implementation of the deep learning model (DLM)

We developed a DLM with 82 convolutional layers and an attention mechanism. The technology details, such as the model architecture, data augmentation, and model visualization, were described previously.14 We used the same architecture to train two new deep learning models for AMI detection and infarct-related artery (IRA) analysis of STEMI. The first deep learning model was trained via full samples with 3 categories, including STEMI, NSTEMI, and not-AMI, and the output of this model was a class-3 softmax output. The second DLM

was trained via STEMI ECGs, and the output of this model was a class-4 softmax output for IRA analysis. The standard input format of the DLM was a length of 1,024 numeric sequences, but the original length of our 12-lead ECG signals was 5,000. In the training process, we randomly cropped a length of 1,024 sequences as input. For the inference stage, 9 overlapping lengths of 1,024 sequences based on interval sampling were used to generate a prediction and were averaged as the final prediction. Due to the scarcity of AMI cases in our study, an oversampling process was implemented to ensure that rare samples were adequately recognized. The settings for the training model were as follows: (1) Adam optimizer with standard parameters ($\beta1 = 0.9$ and $\beta2 = 0.999$) and a batch size of 36 for optimization; (2) a learning rate of 0.001; and (3) a weight decay of 10−4. The 100th epoch model was used as the final model, and the presented performance in the validation set was only evaluated once.

Implementation details of the DLM

(The methodology was fully cited from prior study: EuroIntervention 2021, 17, 765-773.)

The DLM architecture

The architecture of our deep learning model was based on ECG12Net, which was previously used for serum K+ concentration estimation.14 Supposing that a standard 12-lead ECG signal comprised 12 sequences of N numbers (N = 1,250 in our database), the ECG signal sequence X = [x1,1, x1,2, …, x1,N; x2,1, x2,2, …, x2,N; …; x12,1, x12,2, …, x12,N] was used as the input, and the output was a one-hot encoder of AMI categories (STEMI, NSTEMI, and not-AMI) and the IRA of STEMI (STEMI-LMCA, STEMI-LAD, STEMI-LCx, and STEMI-RCA).

For example, a label of STEMI is encoded as [1, 0, 0], and a label of NSTEMI is encoded as [0, 1, 0]. Each output label corresponded to a segment of the input. Because the ECG information was mostly provided by

morphologic changes with shift invariance, convolutional layers with weight sharing were used to adapt to this situation and reduce the hazard of overfitting. We therefore developed a 12-channel sequence-to-sequence model to conduct this task as a revision of DenseNet. The complete architecture of the DLM is shown in **Figure S3**. We defined a "dense unit" as a neural combination as follows: (1) a batch normalization layer to normalize input data, (2) a rectified linear unit (ReLU) layer for nonlinearization, (3) a 1×1 convolution layer with 4K filters to reduce the dimensions of the data, (4) a batch normalization layer for normalization, (5) a ReLU layer for nonlinearization, (6) a 3×1 convolution layer with 4K filters to extract features, (7) a batch normalization layer for normalization, (8) a ReLU layer for nonlinearization, and (9) a 1×1 convolution layer with K filters to extract features. K was a model constant that was set at 32 in all our experiments. After using a dense unit to extract features, we used the dense connectivity resulting from direct connections from any layer to all subsequent layers to build a "dense block." We designed a model with five dense blocks comprising 3, 3, 6, 6, and 3 dense units. Dense blocks cannot be concatenated when the size of the feature maps changes. Thus, a pooling block was used to concatenate each dense block for downsampling in our architecture. This block included a dense unit with a 2×1 stride and an average pooling layer with a 2×1 kernel size and stride, which was used for downsampling. Each dense block was concatenated by the pooling block to integrate the features of the previous blocks. A length of 864 numeral sequences was used as the input in our experiment. We designed an ECG lead block with 80 trainable layers, the architecture of which is shown in Figure S3A. The input data were passed through a batch normalization layer, followed by a convolution layer, another batch normalization layer, a ReLU layer, and a pooling layer. The initial convolution layer comprised K convolution filters with a kernel size of 7×1 and a stride of 2×1. Next, the data were passed through a series of dense blocks and a pooling block, resulting in a 16×1×864 array. A ReLU layer, a batch normalization layer,

and a global pooling layer were followed by the last dense block. Finally, a fully connected layer with k output was created for follow-up use, where k is the number of categories, which was equal to 3 in the first AMI detection model and 4 in the second IRA analysis model of STEMI. This ECG lead block was used to extract 864 features from each ECG lead, making a basic output prediction based on each lead. **Figure S3B** shows how ECG12Net integrated all the information from the ECG to make an overall prediction. ECG12Net comprised 12 ECG lead blocks corresponding to lead sequences. We designed an attention mechanism based on a hierarchical attention network to concatenate these blocks, increasing the interpretive power of ECG12Net. The attention block comprised a batch normalization layer followed by a fully connected layer and then two combinations of a batch normalization layer, a ReLU layer, and a fully connected layer. The first and second fully connected layers each contained 8/k neurons. Attention scores were calculated for each ECG lead and then integrated for standardization by a linear output layer. The standardized attention scores were used to weight the 12 ECG lead outputs by simple multiplication. The 12 weighted outputs were summed and converted into a softmax output layer to provide the final prediction value. The above model using ECG information was named ECG12Net, which contained 82 trainable layers. The m-log-loss function was used to calculate model loss. A dropout layer was added only in the fully connected layer, and the dropout rate was set to 0.5.

Training details

The 12-lead ECG signal sequences were first trained by the 12 ECG leads separately. Due to the seriously uneven distribution in STEMI, NSTEMI, and non-AMI, an oversampling process was implemented to improve performance by ensuring that rare samples were adequately recognized. We sampled 12 STEMI ECGs, 12 NSTEMI ECGs, and 12 not-AMI ECGs in each batch. This process sufficiently considered rare

STEMI and NSTEMI cases so as not to be skewed by the overwhelming number of normal cases. We used the software package MXNet version 1.3.0 to implement ECG12Net. The settings used for the training model were as follows: (1) Adam optimizer with standard parameters (β1 = 0:9 and β2 = 0:999) and a batch size of 36 for optimization; (2) initial learning rate set at 0.001 and lowered by 10 three times when validation loss plateaued after an epoch; and (3) a weight decay of 10−4. Because the sampling rate of our machine is 500 Hz, our 12-lead ECG signal includes 12 numeral sequences with 5,000 digits. However, the standard input format of ECG12Net was a length of 1,024 numeric sequences. We randomly cropped a length of 1,024 sequences as input in the training process. During the inference stage, the 9 overlapping lengths of 1,024 sequences based on interval sampling (X1 to X1024, X498 to X1521, X995 to X2018, X1492 to X2515, X1989 to X3012, X2486 to X3509, X2983 to X4006, X3480 to X4503, and X3977 to X5000) were used to generate predictions and averaged as the final prediction. The 100th epoch model was used as the final model, which presented performance in validation set was only evaluated for once.

Data augmentation

A previous study reported severe overfitting in an atrial fibrillation detection task and suggested a series of data augmentations to improve model performance. In the current study, the problem of overfitting was due to the large number of parameters in the deep learning architecture (~3 million trainable parameters) relative to the sample size. The first step in tackling this issue was to resize the sequence length by adjusting heart rate. We randomly resampled a broader range of heart rates in a uniform distribution from 0.8HR to 1.2HR, where HR was the original heart rate for each sample. The second step was to randomly crop a length of 1,024 sequences as input. The third step was to add a random variable drawn from a Gaussian distribution with a mean of 0 and a standard deviation of 0.1. Fourth, time points were selected uniformly and at random, and the

ECG signal values within a 50 ms vicinity of these points were set at 0. This method was called dropout burst. Finally, we set six random ECG lead sequences to 0 in the combined training step. We observed that the final deep learning model only used information from a few ECG leads to make a prediction and inferred that the model had ceased to learn features from the other ECG leads because it had perfectly predicted all the data in the training set. This approach forced the deep learning model to learn all the abnormal ECG leads.

Model visualization

To interpret the network predictions, we conducted heat maps to visualize the ECG rhythms and leads using class activation mappings (CAMs) and attention mechanisms based on the global average pooling (GAP) architecture in the last network, which was used at the end of each ECG lead. In addition, the various contributions each ECG lead made to the final prediction were weighted by the attention mechanisms, which were used to visualize the importance of each ECG lead.

Automatic active alarm system with notification by short message

The message as shown in **Figure S5,** notified the physician that "An ECG at ED was received for patient X. An ECG indicates high likelihood of STEMI (or NSTEMI with an indicated hsTnI level). Please correlate with clinical condition before final diagnosis." If physicians needed to further identify the ECG, they could click on the link to connect to the ECG immediately (**Figure S5A**), and the results of AI-S prediction were also shown. (**Figure S5B**)

Supplementary Appendix 2. Results

Baseline characteristics of the cohorts

Table S1 shows the patient characteristics in the strategy development and prospective validation cohorts. There were 110 STEMI cases and 125 NSTEMI cases in the strategy development cohort and 59 STEMI cases and 66 NSTEMI cases in the prospective validation cohort. Patients in the strategy development cohort were significantly younger, had higher blood pressure, and had more chronic kidney disease than the prospective validation cohort.

Delayed/misdiagnosed cases by front-line physicians

Four of 59 STEMI (6.8%) cases were delayed diagnoses by front-line physicians, as shown in **Figure 2**. **Figure 2A**: A 36-year-old tall man presented with sudden onset dyspnea and left chest discomfort during exercise. Chest X-ray was performed at first to rule out pneumothorax. **Figure 2B**: An 82-year-old woman presented with sudden onset left shoulder pain. X-ray for shoulder was initially performed. **Figure 2C**: A 75-year-old woman presenting with gastric ulcer receiving esophagogastroduodenoscopy two weeks before this event had worsening epigastric pain, nausea and dizziness. Upright chest X-ray and abdominal X-ray were ordered at first to rule out perforated peptic ulcer. **Figure 2D**: A 54-year-old man with a history of hypertension and chronic kidney disease, stage 3, presented with unresolved chest pain for 3 hours. ECG showed tall T in V2–3, and hyperkalemia could not be ruled out. Lab data showed K^+: 4.8 mmol/L, but hsTnI was 2451 pg/ml.

Supplementary Appendix 3. Supplementary legends to figure

Figure S1. Flow diagram of the generation of strategy development and prospective validation cohorts. Schematic of the data cohort creation and analysis strategy, which was devised to assure a robust and reliable data cohort for strategy development and validation.

Figure S2. Comparison of the STEMI protocol before (A) and after AI-based alarm strategy (B) Details of DtoB time metrics and CCL team action for PPCI in STEMI patients.

Figure S3. Architecture of AI-enable ECG algorithm. A. Electrocardiography (ECG) lead block with 80 trainable layers. B. DLM integrated all the information from the ECG leads to make an overall prediction. The bold and colored words denoted the output dimensions of the layers and the black words signified the important role for the layers. The model constant K was equal to 32 for all the dense blocks and pooling blocks. Conv: convolution; BN: batch normalization; ReLU: rectified linear unit; FC: fully connected. (this figure was fully cited from prior study: *EuroIntervention* 2021, 17, 765-773.)

Figure S4. The rules of AI-based alarm strategy (AI-S). Examples of AMI detected by AI-S during an ED visit. Each data point (ECG or hsTnI) was autouploaded and autodiagnosed by AI-S. The most likely data for AMI were selected for the AI-S algorithm until meeting the alarm operating point. (A) STEMI alarm, (B) NSTEMI alarm

Figure S5. Screenshot of smartphone notification messages triggered by AI-based alarm strategy. (A) Smartphone messages to notify physicians with STEMI patients. (B) ECG with AI prediction result after click on the ECG link.

Figure S6. Not-AMI ECGs with initial detection as STEMI by AI-based alarm strategy (A) and (B) acute perimyocarditis, (C) takotsubo cardiomyopathy, (D) coronary spasm.
