# Supplementary material for: An Artificial Intelligence-Based Alarm Strategy Facilitates Management of Acute Myocardial Infarction
Source: J Pers Med. 2021 Nov 4;11(11):1149. doi: 10.3390/jpm11111149 (PMC8623357; doi:10.3390/jpm11111149)
Supplement: Supplementary file 1 [file jpm-11-01149-s001.zip › Supplementary table S1.pdf]

Supplementary table S1 Corresponding characteristics in development and prospective validation cohorts.

	**Strategy development cohort (n=25,002)**	**Prospective validation cohort (n=14,296)**	**p-value**
**AMI type**			0.821
STEMI	110(0.4%)	59(0.4%)	
NSTEMI	125(0.5%)	66(0.5%)	
not-AMI	24,768(99.1%)	14,171(99.1%)	
**ED information**			
Triage code			0.937
Levels 1 and 2	9,347(37.4%)	5,339(37.3%)	
Levels 3, 4, and 5	15,655 (62.6%)	8,957(62.7%)	
Chest pain			0.928
With	3,320(13.3%)	1,903(13.3%)	
Without	21,682(86.7%)	12,393(86.7%)	
hsTnI test			0.551
With	20,486(81.9%)	11,748 (82.2%)	
Without	4,516(18.1%)	2,548(17.8%)	
**Demorgraphy**			
Gender (male)	12,435(49.7%)	7,239(50.6%)	0.085
Age (years)	61.9±19.9	62.6±19.7	0.001
BMI (kg/m^2)	25.7±118.1	24.7±6.2	0.483
SBP (mmHg)	138.8±27.7	137.2±27.0	<0.001
DBP (mmHg)	79.0±17.6	77.8±17.1	<0.001
**Disease history**			
AMI	848(3.4%)	534(3.7%)	0.075
DM	6,412(25.6%)	3,742(26.2%)	0.250
HTN	9,909(39.6%)	5,666(39.6%)	0.995
HLP	8,055(32.2%)	4,673(32.7%)	0.335
CKD	7,528(30.1%)	4,687(32.8%)	<0.001

Abbreviations: AMI, acute myocardial infarction; STEMI, ST-elevation myocardial infarction; NSTEMI, non ST-elevation myocardial infarction; hsTnI, high sensitivity troponin I; BMI, body mass index; SBP, systolic blood pressure; DBP, diastolic blood pressure; DM, diabetes mellitus; HTN, hypertension; HLP, hyperlipidemia; CKD, chronic kidney disease.
