# Supplementary material for: An Artificial Intelligence-Based Alarm Strategy Facilitates Management of Acute Myocardial Infarction
Source: J Pers Med. 2021 Nov 4;11(11):1149. doi: 10.3390/jpm11111149 (PMC8623357; doi:10.3390/jpm11111149)
Supplement: Supplementary file 1 [file jpm-11-01149-s001.zip › Supplementary table S3.pdf]

Supplementary Table S3 One-year outcomes of study participants after primary percutaneous coronary intervention with door-to-balloon time ≤ 90min.

	**Before AI-S (N=57)**	**After AI-S (N=32)**
All-cause mortality* (CV death)	1(1)	1(1)
HF hospitalization	2	1
Non-fatal MI	2	0

*All-cause mortality included CV death.

Abbreviations: AI-S, AI-based alarm strategy; CV, cardiovascular; HF, heart failure; MI, myocardial infarction.
