# Supplementary material for: An Artificial Intelligence-Based Alarm Strategy Facilitates Management of Acute Myocardial Infarction
Source: J Pers Med. 2021 Nov 4;11(11):1149. doi: 10.3390/jpm11111149 (PMC8623357; doi:10.3390/jpm11111149)
Supplement: Supplementary file 1 [file jpm-11-01149-s001.zip › Supplementaty table S2.pdf]

**Supplementary table S2** Baseline characteristics of study participants before and after AI-S implantation.

| | **Before AI-S (N=57)** | **After AI-S (N=32)** | **p-value** |
|---|---|---|---|
| Age | 59.9±11.9 | 56.2±11.4 | 0.158 |
| Gender(male) | 49(86.0%) | 30(93.8%) | 0.320 |
| BMI | 26.0±3.29 | 25.0±5.98 | 0.342 |
| SBP | 132.2±26.4 | 134.4±34.8 | 0.744 |
| DBP | 81.1±18.1 | 87.4±25.7 | 0.183 |
| Chest pain initially | 55(96.5%) | 29(90.6%) | 0.346 |
| Disease history | | | |
| HTN | 28(49.1%) | 19(59.4%) | 0.353 |
| DM | 24(42.1%) | 10(31.2%) | 0.312 |
| HLP | 25(43.9%) | 20(62.5%) | 0.091 |
| CAD | 8(14.0%) | 3(9.4%) | 0.740 |
| CKD | 8(14.0%) | 6(18.8%) | 0.558 |
| Off hours* | 32(56.1%) | 16(50.0%) | 0.577 |
| Vascular access | | | |
| Transradial | 38(66.7%) | 22(68.8%) | 0.841 |
| Infarct related artery | | | |
| LAD | 32(56.1%) | 22(68.8%) | 0.243 |
| RCA | 19(33.3%) | 7(21.9%) | 0.254 |
| LCx | 6(10.5%) | 2(6.2%) | 0.706 |

*Off hour: from 5:00 p.m. to 8:00 a.m, Monday through Friday; Saturday, Sunday and holidays.

Abbreviations: BMI, body mass index; SBP, systolic blood pressure; DBP, diastolic blood pressure; HTN, hypertension; DM, diabetes mellitus; HLP, hyperlipidemia; CAD, coronary artery disease; CKD, chronic kidney disease; LAD, left ascending artery; RCA, right coronary artery; LCx, left circumflex artery.
