# Supplementary figures and images for: An Artificial Intelligence-Based Alarm Strategy Facilitates Management of Acute Myocardial Infarction
Source: J Pers Med. 2021 Nov 4;11(11):1149. doi: 10.3390/jpm11111149 (PMC8623357; doi:10.3390/jpm11111149)

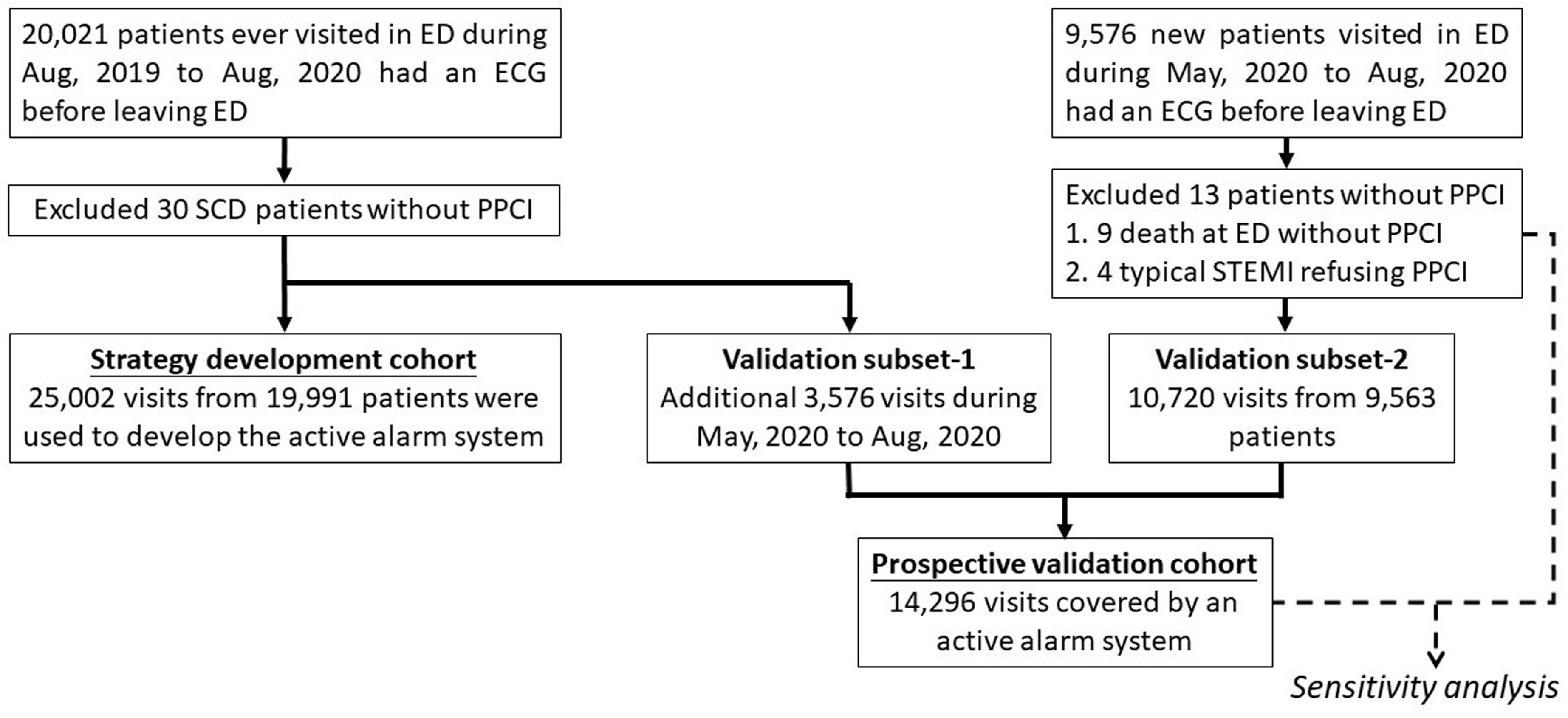

Supplement: Supplementary file 1 [file jpm-11-01149-s001.zip › Supplementary figure S1.jpg]

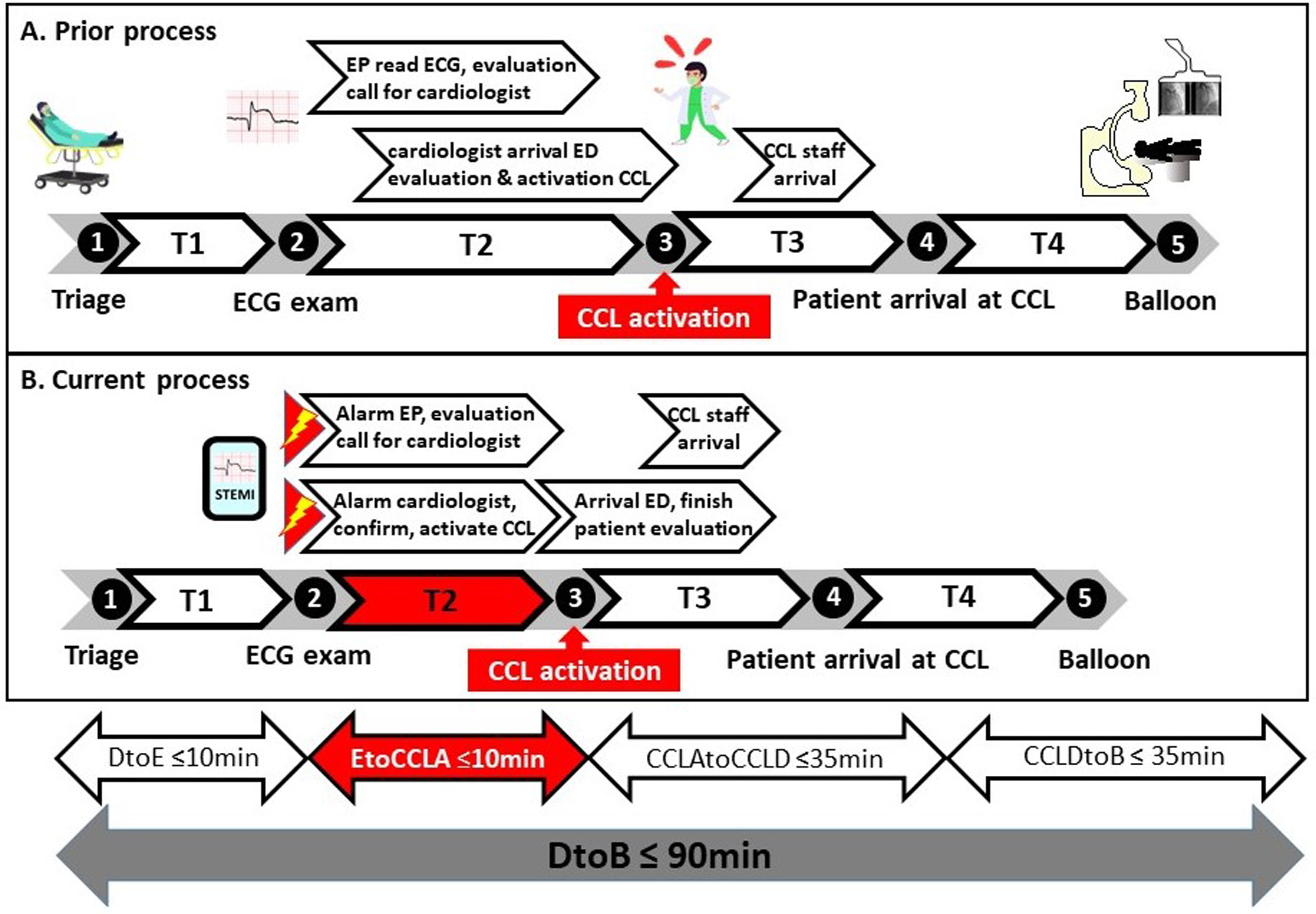

Supplement: Supplementary file 1 [file jpm-11-01149-s001.zip › Supplementary figure S2.jpg]

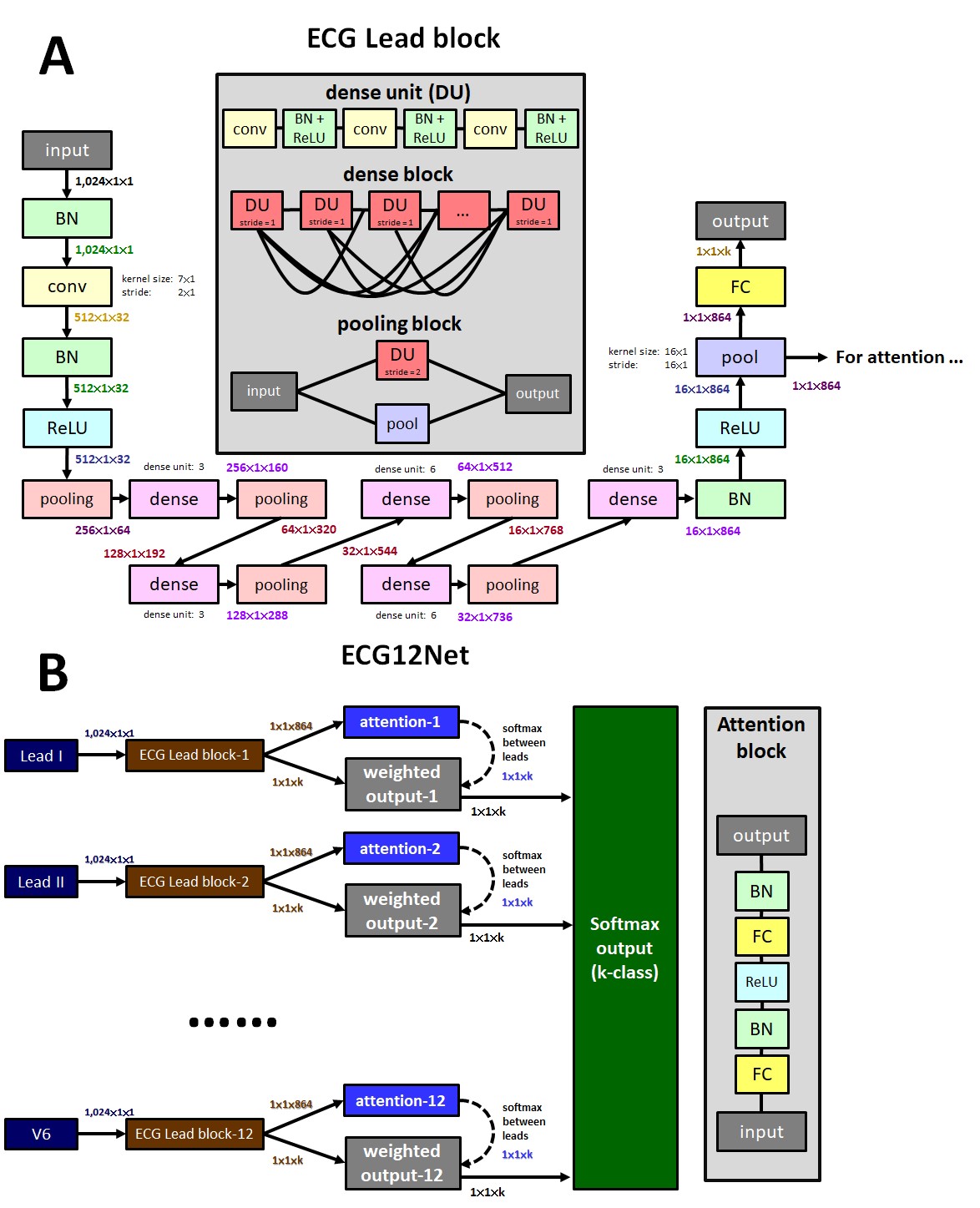

Supplement: Supplementary file 1 [file jpm-11-01149-s001.zip › Supplementary figure S3.jpg]

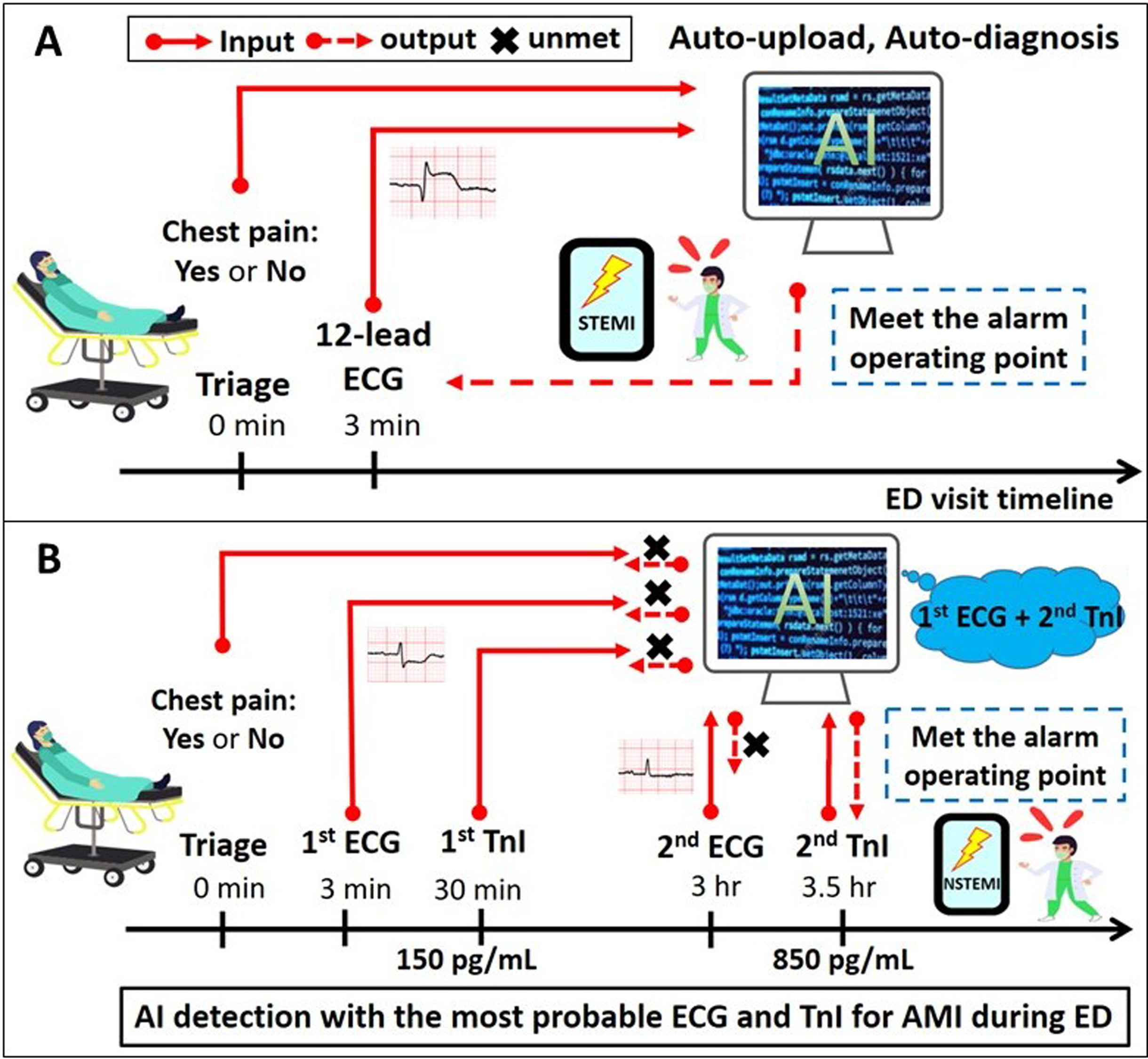

Supplement: Supplementary file 1 [file jpm-11-01149-s001.zip › Supplementary figure S4.jpg]

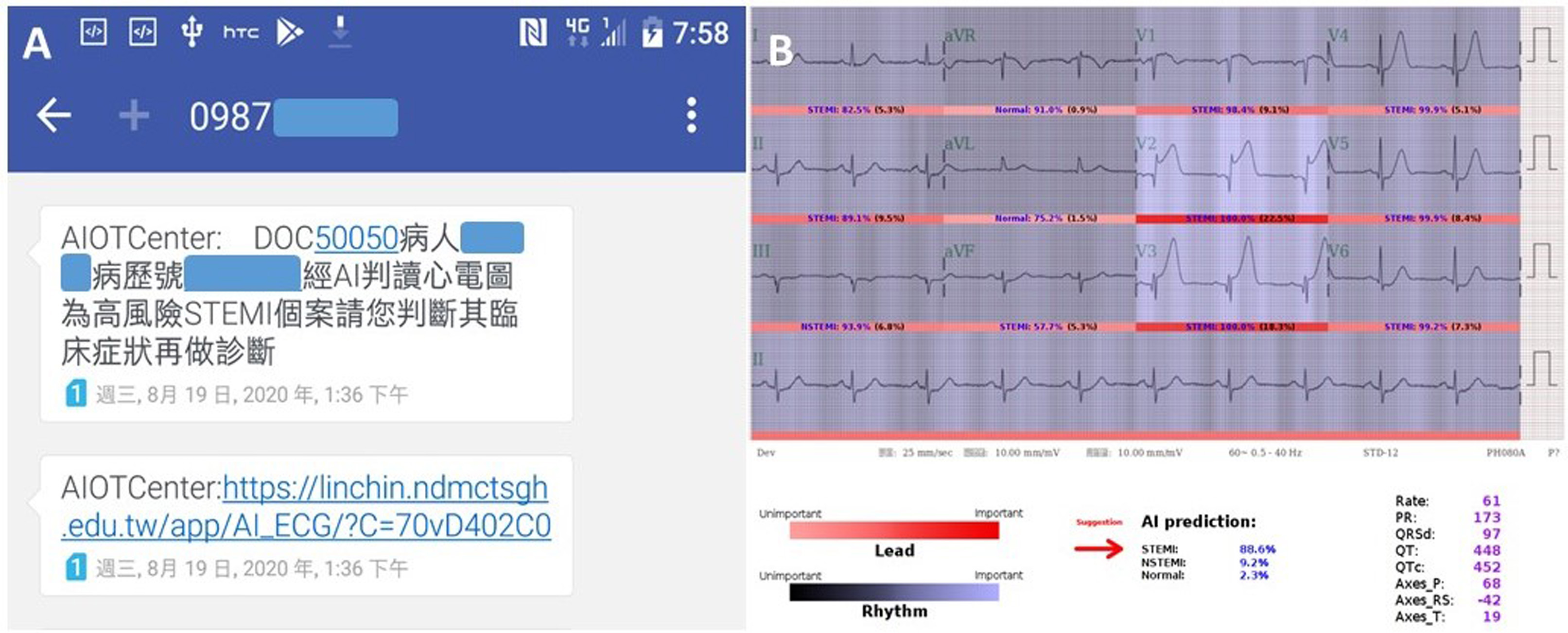

Supplement: Supplementary file 1 [file jpm-11-01149-s001.zip › Supplementary figure S5.jpg]

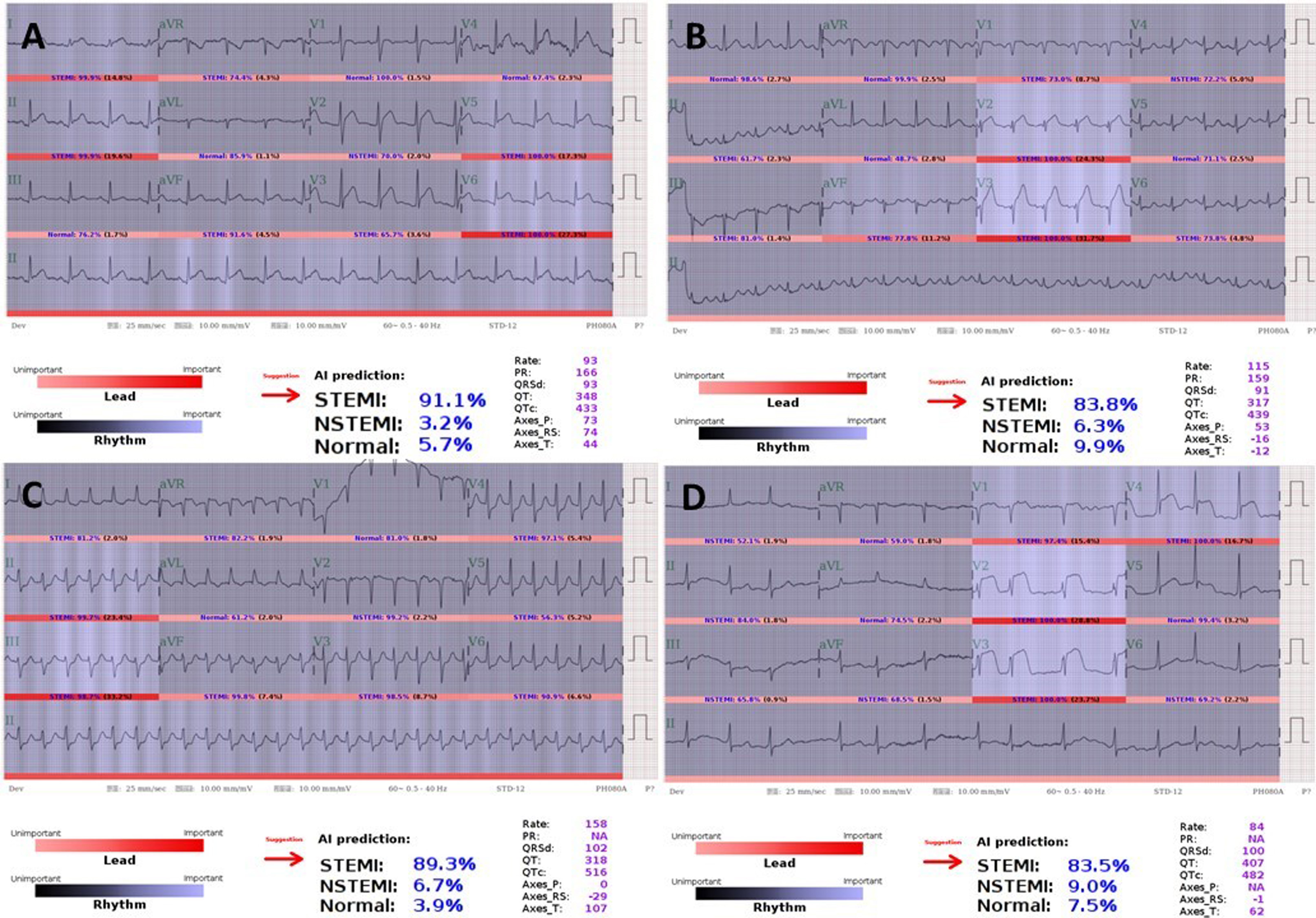

Supplement: Supplementary file 1 [file jpm-11-01149-s001.zip › Supplementary figure S6.jpg]
